# Mammary tumor growth and metastasis are reduced in c‐Kit mutant Sash mice

**DOI:** 10.1002/cam4.696

**Published:** 2016-03-19

**Authors:** Licai He, Zhenfeng Zhu, Shang Chen, Yongping Wang, Haihua Gu

**Affiliations:** ^1^ Key Laboratory of Laboratory MedicineMinistry of EducationSchool of Laboratory Medicine and Life ScienceWenzhou Medical UniversityWenzhou325035China; ^2^ Department of PathologyUniversity of Colorado Anschutz Medical CampusAuroraColorado80045

**Keywords:** Breast cancer, mast cells, metastasis, mouse models, tumor growth

## Abstract

Besides its well‐known function in allergic response, mast cell, one of the key immune cells present in tumor microenvironment, plays important roles in cancer progression. However, the functional role of mast cells in breast cancer development and metastasis is not well understood. To test the involvement of mast cells in breast cancer, we examined the effects of loss of mast cells on mammary tumor development by crossing the well‐known mast cell deficient mouse strain sash (Kit^W‐sh/W‐sh^) with the mammary tumor transgenic mouse strain MMTV‐Polyoma Middle T antigen (PyMT). Although mammary tumor onset was not affected in the absence of mast cells, mammary growth and metastasis were reduced in PyMT/Kit^W‐sh/W‐sh^ mice compared with PyMT/wild‐type mice (WT). Histological and immunofluorescent analyses showed that tumors from PyMT/Kit^W‐sh/W‐sh^ mice showed largely differentiated morphology with reduced angiogenesis compared with MMTV‐PyMT/WT mice. Our results suggest that mast cells may promote breast cancer growth and metastasis. Agents that can block mast cells growth are potential new therapies to treat metastatic breast cancer.

## Introduction

Mast cells are originated from progenitors in bone marrow, travel through the blood, and become differentiated into mature mast cells in various tissues. They are primarily known for their role in allergic responses 1. However, in the last 15 years, studies mainly using mouse models for various types of cancers, including squamous carcinoma 2, neurofibromatosis 3, 4, and pancreatic islet tumor 5, reveal the tumor‐promoting role of mast cells in different cancers. Meanwhile, the suppressive function of mast cells in cancer was implicated in a few studies, including in intestinal tumorigenesis 6. Most of these studies used mutant mice strains that are deficient in mast cells. These mutant mice include the Kit^W/Wv^ 7 and Sash (Kit^W‐sh/W‐sh^) strains 8, which have mutations in the coding regions that impair the kinase activity of c‐Kit, and in the 5′ untranslated region that inhibits c‐Kit expression, respectively. The main function of mast cells in tumor stroma is to stimulate the tumor cell growth directly or indirectly by promoting tumor growth and angiogenesis through secreting factors and proteases during the premalignant stage.

The roles of mast cells in breast cancer have been controversial. Mast cells are found in the stroma of human mammary carcinoma 9, 10. However, the results from various studies examining the association of mast cells with disease outcome are conflicting. It has been reported that the presence of higher number of mast cells in breast tumor stroma is correlated with good prognosis of the patients 11, 12. Another study has shown higher mast cell number in sentinel lymph node is correlated with increased angiogenesis and micrometastases in patients with breast cancer 13. Thus, it is not clear whether mast cells play a positive or negative role during breast cancer progression and metastasis.

To clarify the role of mast cells in mammary tumor development and metastasis, we examined the effects of loss of mast cells on mammary tumor progression by crossing the mast cell deficient mice Kit^W‐sh/W‐sh^ with the MMTV‐Polyoma Middle T antigen (PyMT) mice, which is a well‐known transgenic mouse model of breast cancer that develops pulmonary metastasis 14. We found that mammary growth and metastasis are reduced in PyMT/Kit^W‐sh/W‐sh^ mice compared with PyMT mice, suggesting that mast cells play a positive role in mammary development and metastasis.

## Materials and Methods

### Mice and genotyping

MMTV‐PyMT (PyMT) mice, originally generated by Bill Muller's lab 14, in C57BL/6 background were kindly provided by Dr. Sandra Gendler (Mayo Clinic, Arizona). Sash (Kit^W‐sh/W‐sh^) mice in C57BL/6 were purchased from The Jackson Laboratory (Bar Harbor, ME). Male PyMT mice were mated with female Kit^W‐sh/W‐sh^ mice to generate progenies with the genotype of Kit^+/W‐sh^ or PyMT (PyMT+/−)/Kit^+/W‐sh^. Female Kit^+/W‐sh^ and male PyMT/Kit^+/W‐sh^ mice were mated against each other to generate female littermates that are PyMT/wild‐type (WT) and PyMT/Kit^W‐sh/W‐sh^. Mice were genotyped for the PyMT allele by polymerase chain reaction (PCR) using the following primers: forward primer, AGTCACTGCTACTGCACCCAG‐and reverse primer, CTCTCCTCAGTTCTTCGCTCC as described 15. Sash mutant mice were genotyped based on the coat color that shows mice with black fur with white sash at the midline (The Jackson Laboratory). Protocols for all mouse experiments were approved by the Institutional Animal Care and Use Committee at the University of Colorado Anschutz Medical Campus and Wenzhou Medical University.

### Monitoring mammary tumor development

At 60 days of age, mice were palpated every week to detect the onset of mammary tumor development. After the detection of palpable tumors, mice were monitored for tumor growth with caliper every 2 weeks. Total tumor volume was determined using the formula: volume = (L*W^2^)/2. Mice were killed at 8 weeks or 10 weeks after the onset of tumors.

### Quantitation of lung metastasis

Lung tissues were dissected from mice, flash frozen in liquid nitrogen, and stored at −80°C. Total RNAs (1 *μ*g) isolated from lungs using Trizol Reagent (Invitrogen, Carlsbad, CA) and tissue homogenizer (Kinematica Inc, Bohemia, NY) were treated with DNaseI and reverse transcribed into cDNA using Superscript III (Invitrogen). Quantitative Reverse Transcription (RT)‐PCR was performed on 7500 fast real‐time PCR systems (Applied Biosystems, Foster City, CA) using Power SYBR Green PCR Master Mix (Applied Biosystems). PCR primers were: PyMT gene, forward primer, AGCCCGATGACAGCATATCC, and reverse primer, GGTCTTGGTCGCTTTCTGGAT; mouse GAPDH gene, forward primer, AGGTCGGTGTGAACGGATTTG, Reverse Primer: TGTAGACCATGTAGTTGAGGTCA. Relative PyMT mRNA level normalized to GAPDH mRNA level represents the relative lung metastasis of the PyMT mammary tumors.

### Histology and immunohistochemistry analyses

Tumor and lung tissues were fixed in 10% formalin or Bouin's solution for 24 h, and embedded in paraffin. Sections were stained with Hematoxylin and Eosin (H&E) or toluidine blue to visualize mast cells as described 16. For immunochemistry (IHC), paraffin sections were deparaffinized, and antigens were heat retrieved in 10 mmol/L citrate buffer pH 6.0 in microwave. Anti‐CD31/PECAM1 rabbit antibodies were purchased from Santa Cruz Biotechnology (SC‐1506). Envision+ anti‐rabbit horseradish peroxidase system (Dako, Carpinteria, CA) was used for antibody detection. CD31‐stained images were captured under the 10X objective lens using the Nikon Eclipse Ti‐S/L 100 microscope system. Images of six random fields for each tumor were taken as TIF files. The optical intensity of CD31 IHC staining in each field was analyzed and normalized to the area size of the chosen field using image‐pro plus software.

### Statistical analysis

Survival curves were generated using the Kaplan–Meier method, and significance was evaluated using the log‐rank test. Paired data were evaluated by unpaired two‐tailed Student's *t* test.

## Results

To explore the function of mast cells during mammary tumor progression, we examined the effects of loss of mast cells on mammary tumor development by crossing the mast cell deficient mice sash (Kit^W‐sh/W‐sh^) (C57BL/6 background) with the MMTV‐PyMT mice (C57BL/6). Kit^W‐sh/W‐sh^ mice have a DNA inversion mutation in the 5′ upstream regulatory region of the c‐Kit gene that results in impaired c‐Kit expression and mast cell development in tissues 8. Compared to the widely used mast cell deficient mice strains such as Kit^W/Wv^, Kit^W‐sh/W‐sh^ mice are fertile and without anemia, and have pretty normal development of other hematopoietic lineages such as lymphocytes and macrophages 8. Tumor development in MMTV‐PyMT(PyMT) mice follows a progression path including hyperplasia, adenoma, early carcinoma and late carcinoma, which is very similar to progressions seen in human breast cancers 17. PyMT mice in the C57BL/6 background develop palpable breast tumors around 90 days of age 15, which is slower than mice in the FvB background 14.

**Figure 1:**
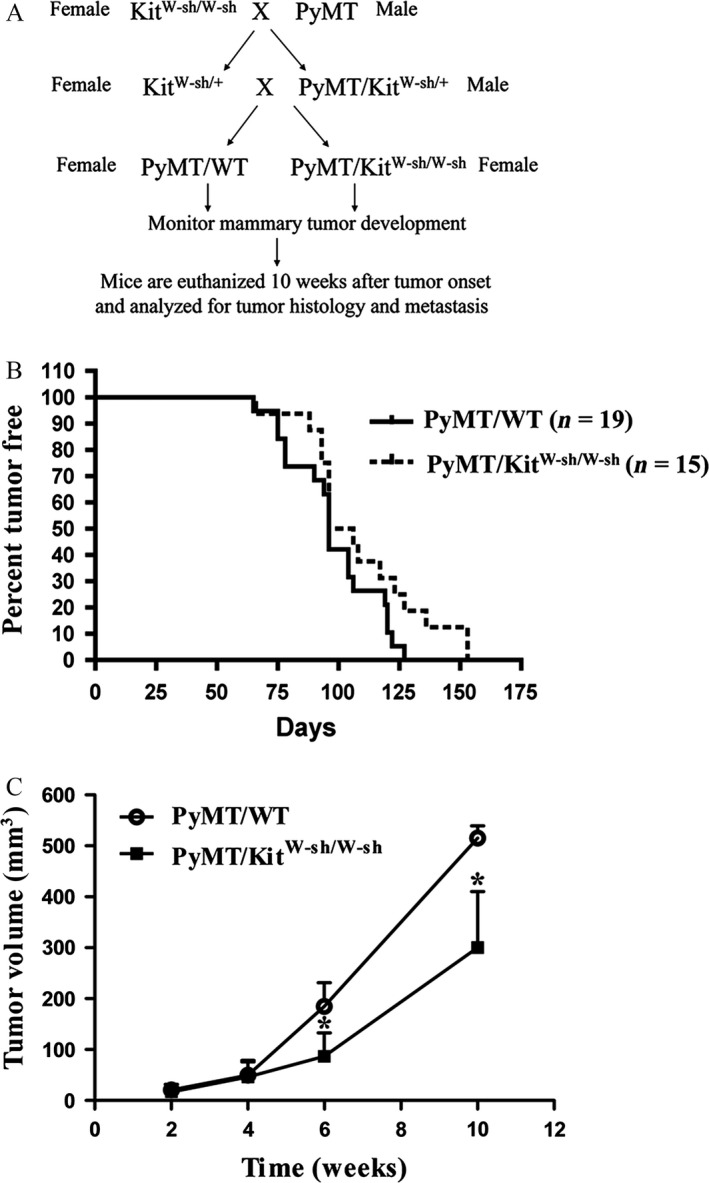
Loss of mast cells reduces mammary tumor growth. (A) Breeding and experimental Scheme. (B) Mammary tumor onset is similar between PyMT/WT and PyMT/Kit^W‐sh/W‐sh^ female mice. Nineteen PyMT/wild‐type (WT) mice (*n* = 19) and 15 PyMT/Kit^W‐sh/W‐sh^ mice (*n* = 15). *P* = 0.54. (C) Mammary tumor growth is reduced in PyMT/Kit^W‐sh/W‐sh^ mice compared with PyMT/WT mice. After palpable tumors were detected, four mice (*n* = 4) for each group were monitored for their tumor growth with digital caliper for 10 weeks. *P < 0.05.

According to the breeding strategy illustrated in Figure 1A, we generated female control PyMT/WT and littermate PyMT/Kit^W‐sh/W‐sh^ mice. Mice with both genotypes developed palpable mammary tumors between 60 and 150 days of age (Fig. 1B). Mammary tumor onset of PyMT/Kit^W‐sh/W‐sh^ mice is not significantly different from that of the control PyMT/WT mice. This result indicates that mast cells are not required for the induction of mammary tumors. Upon detection of tumor onset, four PyMT/WT and 4 littermate PyMT/Kit^W‐sh/W‐sh^ mice were monitored for tumor growth biweekly for 10 weeks.

The growth rate of the PyMT/Kit^W‐sh/W‐sh^ tumor is about 50–60% of PyMT/WT tumors (Fig. 1C).

**Figure 2:**
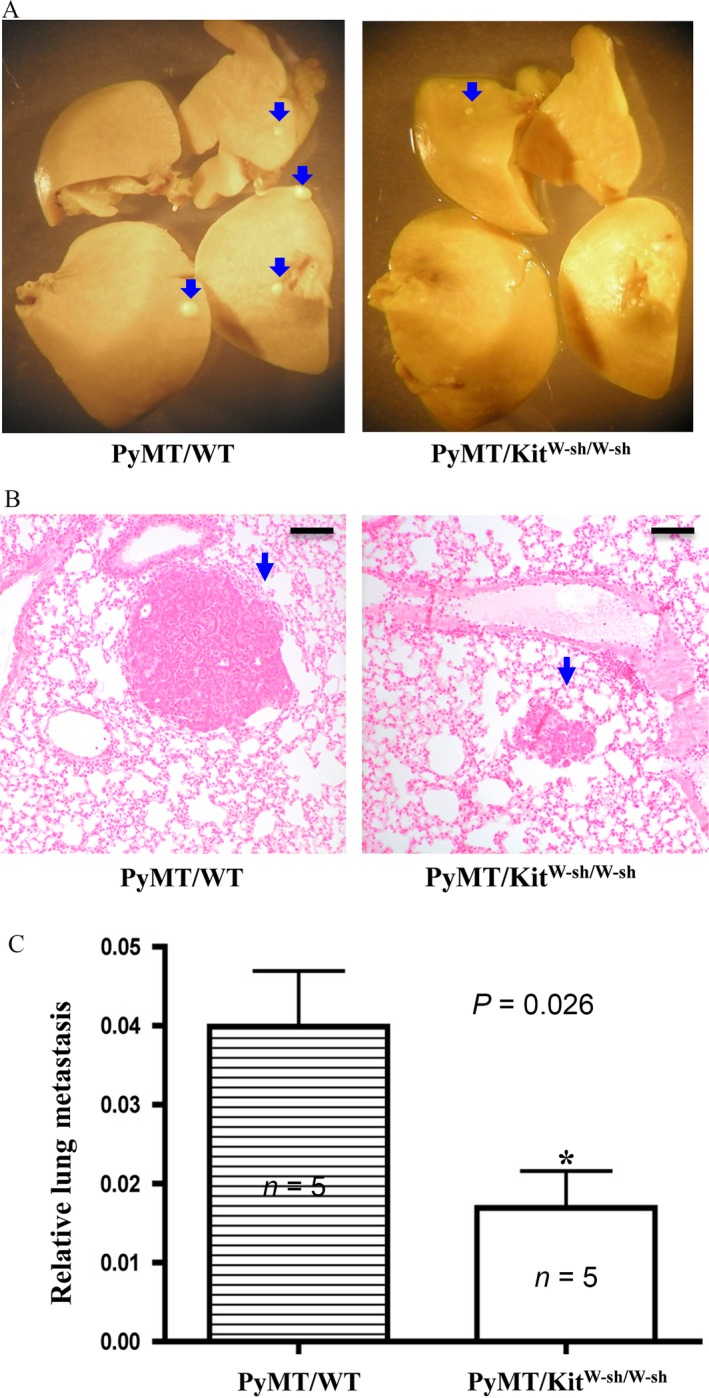
Loss of mast cells impairs lung metastasis of mammary tumors. (A, B) The number of metastasis foci is reduced in PyMT/Kit^W‐sh/W‐sh^ mice compared with PyMT/WT mice. Lungs were dissected out, fixed in Bouin's solution, and photographed (A), sectioned and stained with H&E (B). Blue arrows indicate the metastatic foci. Bar = 200 *μ*m. (C) Quantitation of lung metastasis. Lungs were dissected from mice, and homogenized in Trizol solution to isolate total RNAs. Real‐time RT‐PCR was used to quantitate the relative amount of PyMT mRNA levels (normalized to GAPDH RNA level), which reflects the relative PyMT lung metastasis. Five mice for each group (*n* = 5), *P* = 0.026.

Ten weeks after the detection of palpable tumors, the mice were euthanized and lung tissues were dissected out for examination of metastasis. Lung metastasis foci were visually seen in PyMT/WT mice. In contrast, lung metastasis foci were significantly reduced both in size and number in PyMT/Kit^W‐sh/W‐sh^ mice (Fig. 2A and B). Quantitation of lung metastasis by measuring the relative amounts of PyMT mRNA present in the lungs showed that the lung metastasis was reduced by more than 50% in PyMT/Kit^W‐sh/W‐sh^ mice compared with PyMT/WT mice (Fig. 2C).

**Figure 3:**
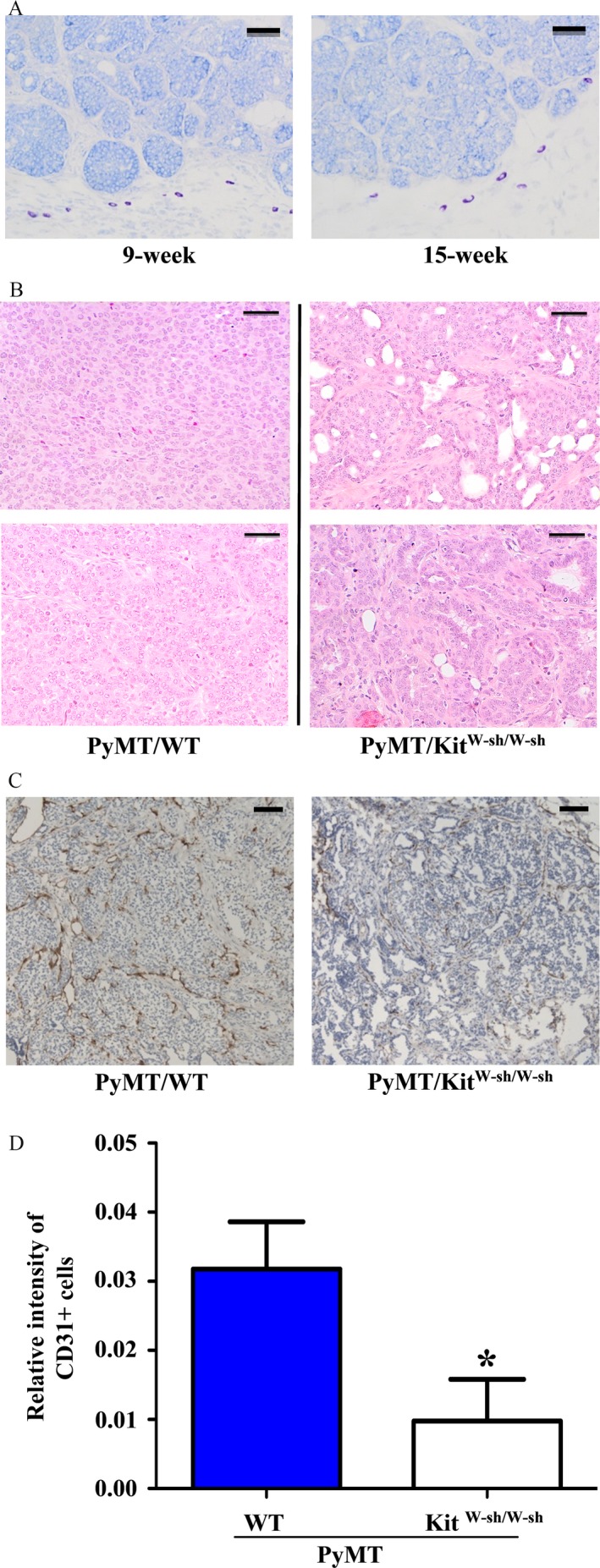
Histological and immunohistochemistry analyses of mammary tumors. (A) Mast cells are present in the stroma of mammary tumors. Mammary tumors from 9 (left) and 15 (right) week‐old female PyMT/WT mice were dissected out, fixed, embedded, sectioned, and stained with Toluidine blue. Dark purple‐stained cells are mast cells. Bar = 100 *μ*m. (B) Hematoxylin and eosin (H&E) staining of mammary tumors from PyMT/WT and PyMT/Kit^W‐sh/W‐sh^ female mice (10 weeks after tumor onset). Tumors from 2 PyMT/WT control mice displayed poorly differentiated morphology (left). In contrast, breast tumors from 2 PyMT/Kit^W‐sh/W‐sh^ mice showed more differentiated property (right). Bar = 100 *μ*m. (C, D) Angiogenesis is impaired in tumors of PyMT/Kit^W‐sh/W‐sh^ mice. (C) Paraffin sections of tumors (2 months after tumor onset) from PyMT/WT and PyMT/Kit^W‐sh/W‐sh^ mice were stained with anti‐CD31 antibody. Bar = 200 *μ*m. The dark brown colors are the CD31‐stained blood vessels. (D) The area stained by anti‐CD31 antibody was analyzed by the Image‐pro plus software as described in the Materials and Method section. Result shown is the average of relative CD31^+^ signal per microscope field from three tumors for each group. *P < 0.05.

The presence of mast cells in mammary tumors from PyMT/WT and PyMT/Kit^W‐sh/W‐sh^ mice were examined by toluidine blue staining. Mast cells were found around the edge of mammary tumors in PyMT/WT mice but not inside the mass of mammary tumor cells (Fig. 3A). As expected, no mast cells were detected in any parts of the mammary tumors from PyMT/Kit^W‐sh/W‐sh^ mice (data not shown). These results strongly suggest that mast cells play a positive role in breast cancer progression.

The histology of the mammary tumors was examined by H&E staining from mice killed 10 weeks after the onset of tumor. Tumors in PyMT/WT mice were mainly poorly differentiated and with solid sheets of malignant cells (Fig. 3B left). In contrast, large portion of the tumors in PyMT/Kit^W‐sh/W‐sh^ mice showed well‐differentiated properties (i.e., the presence of the basement membranes with luminal space) (Fig. 3B right).

Because mast cells promote tumor growth via increased angiogenesis in several mouse tumor models (30, 31), tumor angiogenesis was also examined in tumor sections by immunohistochemistry with the anti‐CD31 antibodies that stain endothelial cells in blood vessels. We found that there was ~70% decrease in the density of CD31‐stained blood vessels in PyMT/Kit^W‐sh/W‐sh^ tumors compared with that in PyMT/WT tumors (Fig. 3C and D). These results suggest that mast cells may be important for angiogenesis of mammary tumors.

## Discussion

Our study is the first one that applies the widely used mast cell deficient mouse strain.

Sash (Kit^W‐sh/W‐sh^) to test the role of mast cells in mammary tumor development. The results of our study suggest that mast cells play positive roles in the growth and metastasis of breast cancer.

Published studies imply that mast cells promote metastasis of breast cancer. Our study provides the functional data to support the important role of mast cells in metastasis of mammary tumors. Examining mast cells in human breast tumor samples showed that higher mast cell number in sentinel lymph node is correlated with increased angiogenesis and micrometastases in patients with breast cancer 13. A recent study revealed that arthritis promotes mammary tumor metastasis to lung through mast cells in the MMTV‐PyMT mouse model 18. Treating mice with an antibody against c‐Kit reduced mast cells and lung metastasis in this arthritis model 18. Our observations that reduced lung metastasis and tumor angiogenesis in PyMT/Kit^W‐sh/W‐sh^ mice are consistent with these published results.

Because mast cells are mainly present at the peripheral sites of breast tumors 9 (Fig. 3A), it is very possible that mast cells secrete factors or proteases that help breast cancer cells break away from their primary site.

The result of our study is also inconsistent with the conclusions from several published reports. Some published literatures revealed breast cancer with high levels of mast cells present in tumor stroma is associated with better outcome 9, 10. Gene expression study has shown that MMTV‐PyMT mammary tumor model resembles luminal breast cancer more closely 19. It is possible that mast cells may have different functions in the luminal (ER+) breast cancer compared with Her2+ and triple negative breast cancer. Alternatively, mast cells may have different functions at earlier and later stages of breast cancer development. Data in our model strongly suggest that the early presence of stromal mast cells promotes mammary tumor growth and metastasis. However, in human breast cancer, the presence of higher numbers of mast cells in the fully developed breast tumors may impede breast cancer progression.

Our result indicates that mast cells do not affect mammary tumor onset. Interestingly, the result from an animal study using the Ws/Ws rats, deficient in mast cells in vivo, in response to N‐nitrosomethylurea (NMU) treatment seems to suggest that mast cells inhibit the onset of mammary tumors induced by NMU 20. In the Ws/Ws rat study, rats are from mixed background of Brown Norway and Donryu strains, and the control WT rats are not littermates, which may contribute to their different sensitivity to NMU induced mammary tumorigenesis. In contrast, mice are littermates in C57BL/6 background in our study. In addition, mammary tumor growth and metastasis were not examined in the Ws/Ws rat study 20.

Published studies, together with our data, suggest that mast cells may promote tumor angiogenesis that is important for mammary tumor metastasis. Our study showed that there was reduced tumor angiogenesis in PyMT/Kit^W‐sh/W‐sh^ mice compared with PyMT/WT mice (Fig. 3C). In the MMTV‐PyMT model, it has been demonstrated elegantly that inhibition of the angiogenic switch 21, due to the loss of macrophages‐derived angiogenic factor VEGF 22, blocks lung metastasis of mammary tumors. Studies from the squamous carcinoma mouse model indicate that mast cells‐derived matrix metalloprotease 9 (MMP9) is important for the production of mature VEGF in the tumor microenvironment 23. Future studies are required to ascertain whether mast cells regulate the production of MMPs or some factors to promote mammary tumor angiogenesis and metastasis in our model. In addition, future experiment involving reconstitution of Kit^W‐sh/W‐sh^ mice with WT mast cells should also help exclude whether other types of hematopoietic cells contribute to the phenotypes that we described in our model.

In conclusion, data from our study using mouse models provide strong functional evidence to support a positive role of mast cells in breast cancer growth and metastasis. Drugs or agents that can inhibit the growth of mast cells may be useful to treat patients with metastatic breast cancer in the future.

## Conflict of Interest

None declared.
